# Chalcogen Doping in SnO_2_: A DFT Investigation of Optical and Electronic Properties for Enhanced Photocatalytic Applications

**DOI:** 10.3390/ma17163910

**Published:** 2024-08-07

**Authors:** Nikolaos Kelaidis, Yerassimos Panayiotatos, Alexander Chroneos

**Affiliations:** 1 Theoretical and Physical Chemistry Institute, National Hellenic Research Foundation, Vass. Constantinou 48, 11635 Athens, Greece; 2 Department of Mechanical Engineering, University of West Attica, 12241 Athens, Greece; gpana@uniwa.gr; 3 Department of Electrical and Computer Engineering, University of Thessaly, 38221 Volos, Greece; 4 Department of Materials, Imperial College, London SW7 2AZ, UK

**Keywords:** SnO_2_, electronic properties, optical properties, density functional theory

## Abstract

Tin dioxide (SnO_2_) is an important transparent conductive oxide (TCO), highly desirable for its use in various technologies due to its earth abundance and non-toxicity. It is studied for applications such as photocatalysis, energy harvesting, energy storage, LEDs, and photovoltaics as an electron transport layer. Elemental doping has been an established method to tune its band gap, increase conductivity, passivate defects, etc. In this study, we apply density functional theory (DFT) calculations to examine the electronic and optical properties of SnO_2_ when doped with members of the oxygen family, namely S, Se, and Te. By calculating defect formation energies, we find that S doping is energetically favourable in the oxygen substitutional position, whereas Se and Te prefer the Sn substitutional site. We show that S and Se substitutional doping leads to near gap states and can be an effective way to reduce the band gap, which results in an increased absorbance in the optical part of the spectrum, leading to improved photocatalytic activity, whereas Te doping results in several mid-gap states.

## 1. Introduction

SnO_2_ is an n-type wide band gap (~3.6 eV) semiconductor that has attracted much interest in the scientific community due its abundance, non-toxicity, and interesting and tunable properties. Potential applications include gas sensing [1,2], photocatalysis [3], energy storage [4], and use as a transparent conducting oxide (TCO) in touchscreens, flat-panel displays, and photovoltaic cells. Various new and efficient preparation methods [5] have been developed for SnO_2_ growth, such as magnetron sputtering [6] and electrochemical deposition [7], which are expected to facilitate scalability and lead to large-area, uniform SnO_2_ films. Although undoped SnO_2_ has a rather wide band gap and primarily absorbs shortwave ultraviolet light, this can be addressed by tuning its properties through established methods such as varying its morphology [8,9] and introducing doping [10,11]. The modification of band gaps and band edges has been a matter of research, particularly from a computational perspective [12,13].

Here, we investigate the impact of doping with the chalcogen elements S, Se, and Te. S, Se, and Te (though mainly S) have been examined for sensing applications, but it is also interesting to investigate the effect of their doping on optical properties when they are used in optoelectronics or as a transport layer in photovoltaics, given the limited theoretical studies on these dopants.

Experimental studies have shown that doping with S (as a surface dopant) significantly improves the sensitivity of resistive NO₂ gas sensors [14]. In the experimental study by Ma et al. [15], S-doped SnO_2_ nanoparticles were successfully prepared and characterised. These nanoparticles exhibited increased photocatalytic activity due to a significantly improved visible light response, leading to the effective separation of photo-generated electron–hole pairs.

In a recent computational study, Yu et al. [16] calculated the effect of non-metal doping of SnO_2_, including sulphur. They modelled S at an oxygen substitutional position and used the PBE functional to calculate the electronic properties and band gap. The calculated band gap in this study was 1.28 eV, and the effect of S was examined, mainly being the introduction of impurity levels into the band gap.

The effect of Se as a dopant has been also examined for sensing applications [17]. Se-doped SnO_2_ was found to exhibit a 2 to 3 times higher response towards certain gases attributed to a considerable electron transfer by the dopant Se, which was found to be prone to act as a substitutional dopant on the Sn site. DFT calculations were performed on Sn_16_O_32_ slabs to examine the activation energy barrier of the CO oxidation, which was found to be significantly lower for Se-doped than pristine SnO_2_. The density of states was computed with the PBE functional.

Regarding Te as a dopant, a recent study [18] prepared Sn-O-Te layers and examined them as humidity sensors. The preparation method involved thermal co-evaporation of Sn and TeO₂ in a vacuum, resulting in a nanosized SnO₂ matrix with a finely dispersed phase of Sn, Te, TeO₂, and SnTe. Elemental Te exhibits high mobility and begins to diffuse from the bulk and segregate on the layer surface at temperatures above 100 °C. At room temperature, as-deposited layers with Sn/Te ratios between 0.4 and 0.9 show excellent characteristics as humidity sensors. It was determined that both electron and ionic conduction simultaneously drive the overall conduction mechanism of the sensor response.

A large-scale computational work [19] examined the screening of a total of 63 SnO_2_ dopants, all positioned in a Sn substitutional site, including S, Se, and Te. PBE calculations were applied as a screening method. The importance of a high level of theory, such as the PBE0 hybrid functional for the calculations of the electronic properties of defected structures, was pointed out. The authors concluded that p-type doping is not possible via elemental substitution of Sn. Nevertheless, that study was not extended to oxygen substitutional or interstitial dopants.

In the present study, we employ DFT calculations to examine the electronic, structural, and optical properties of SnO_2_ doped with S, Se, and Te at both oxygen or sulphur substitutional and interstitial sites, which seems to be lacking in the literature. The electronic and optical properties of doped SnO_2_ are systematically examined with a higher level of theory, employing hybrid functionals. Additionally, we have employed a ‘harder’ Sn projector wave potential (Sn_d) that provides higher accuracy and correctly predicts the SnO_2_ band gap. We believe that this study can be of interest for potential application in solar cells and photocatalysis.

## 2. Materials and Methods

**Figure 1:**
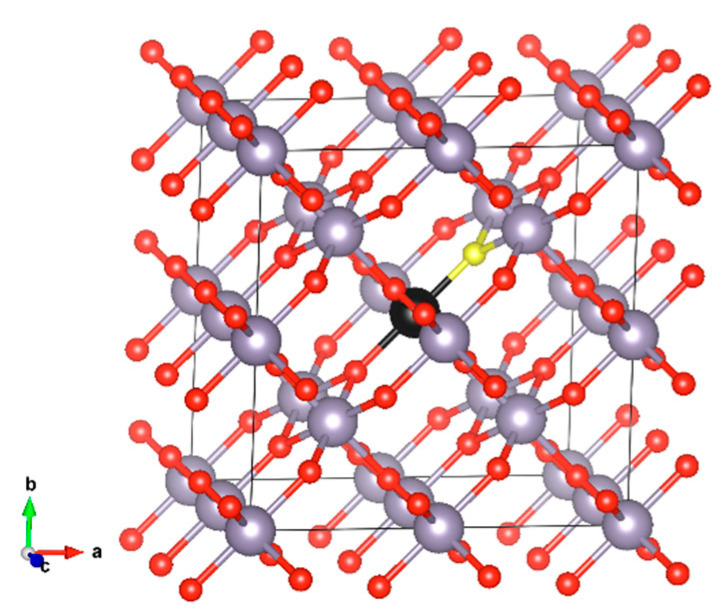
SnO_2_ 2 × 2 × 2 supercell (a) initial (perfect) structure. Sn occupies the 2a Wyckoff site and O occupies the 4f site. The S atom that is substituted in our model (black) and the oxygen atom (yellow) are shown in different colours.

The crystal structure of SnO_2_ is that of rutile. It crystallises in the tetragonal P4₂/mnm space group with the lattice constants of a = 4.737 Å and c = 3.186 Å, as determined by X-ray diffraction (XRD) experiments [20]. For the defect calculations, we used a 2 × 2 × 2 supercell approximation, which consists of 48 atoms in the perfect cell configuration (see Figure 1). We substituted either the Sn atom (for substitutional cation doping) or the O atom (for substitutional anion doping) with members of the chalcogen group (excluding polonium), namely S, Se, and Te. All Sn positions in the crystal structure are equivalent and occupy the 2a Wyckoff site, as do all oxygen positions, which occupy the 4f Wyckoff site. Additionally, we examined these dopants at interstitial positions. To account for size-related errors, we also performed calculations using a larger 3 × 3 × 3 supercell [21]. However, when comparing defect formation energies with those from the 2 × 2 × 2 supercell, the differences were minimal, on the order of about 0.1 eV.

The calculations were performed with the aid of the Cambridge Serial Total Energy Package (CASTEP) code using the PBE functional. Following the convergence tests, 800 eV was chosen as the cutoff energy and a Brillouin zone 3 × 3 × 3 k-point sampling was chosen for the geometry optimisation. In order to account for the impact of localised electrons and the underestimation of the band gap that is typically observed in GGA and LDA, we applied the hybrid functional PBE0 [22] for the electronic and optical property calculations applied on the 2 × 2 × 2 supercell with a denser k-point mesh of 5 × 5 × 5. Regarding convergence criteria for our calculations, the self-consistent field (SCF) method tolerance was 1.0 × 10^−5^ eV/atom, the force tolerance was set at 0.05 eV/Å, and the max displacement tolerance was set at 0.001 eV/Å.

## 3. Results and Discussion

### 3.1. Formation Energies and Structural Optimisation

For the calculation of the formation energies for the various doping cases, geometry optimisation was performed using the PBE functional. The following formula was applied for the calculation of the defect formation energy:

(1) $$E_{d}^{f}=E_{T}^{defect}-E_{T}^{perfect}+\underset{i}{\sum }\mu_{i}n_{i}$$

where $E_{d}^{f}$ is the defect formation energy, $E_{T}^{perfect}$ is the total energy of the supercell with no defects, and $E_{T}^{defect}$ is the total energy of the supercell where *n_i_* atoms of chemical potential *μ_i_* have been added or removed.

Since we are examining doping with a single element, this formula can be reduced to a simpler form:

(2) $$E_{formation}=E_{perfect}-E_{doped}-\left(\mu_{dopant}+\mu_{substituted}\right)$$

The chemical potential of each element was determined by calculating the total energy per atom in its most stable, standard state. The calculated defect formation energies are presented in Table 1, along with the DFT calculated lattice constants, cell volume, and relative volume expansion.

An important conclusion from the calculation of the defect formation energies is that doping with S appears energetically favourable on the oxygen site, although it is found to be so in the Sn position in some computational studies. Here, we find that the formation energy of S as an oxygen substitutional atom is 3.95 eV, which is significantly less than that at the Sn substitutional position, which is 5.43 eV. We also find that Se and especially Te are preferably Sn-substitutional dopants, with a formation energy of 3.90 eV for Se (less than the 5.04 eV at the oxygen substitution site) and just 2.88 eV for Te (7.66 eV at the oxygen substitutional position, which renders this case unfeasible).

**Figure 2:**
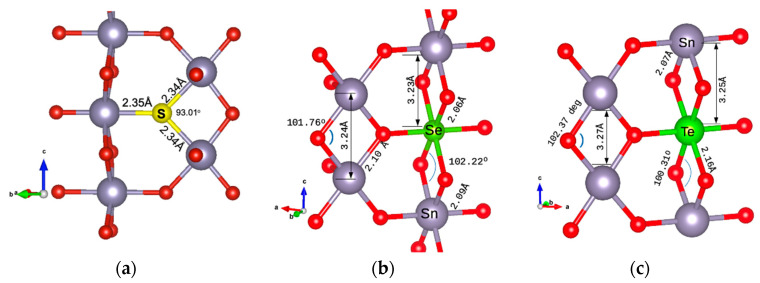
Dopant positions in the SnO_2_ 2 × 2 × 2 supercell of (**a**) S substituting an oxygen atom, (**b**) Se substituting a Sn atom, and (**c**) Te substituting a Sn atom.

The minimum energy configuration structures are shown in Figure 2 for the energetically preferable doping cases. In Figure 2a, the structure with S as an oxygen substitution is presented. The Sn-S atomic distances are calculated as 2.34 Å. In Figure 2b, the case of doping with Se as a Sn substitution is shown. The Se-O bond is at 2.064 Å, slightly smaller than the Sn-O bond, which is at 2.098 Å. The Se-Sn bond is at 3.233 Å, very similar to the Sn-Sn bond (calculated for this structure as 3.236 Å). In Figure 2c, the Te atom is at a Sn substitutional site. The Te-O distance is calculated as 2.16 Å and the Te-Sn distance as 3.253 Å, the same as the Sn-Sn bond length. Structures were drawn using VESTA visualisation software (version 3) [23].

In Table 1, the relative volume change is shown. The substitution of Sn with S is the only case of a decrease in total volume, attributed to the smaller atomic radius of S compared to Sn (100 pm vs. 140 pm). When substituting oxygen with S, Se, or Te, the total cell volume is increased. When dopants are located in an interstitial position, the cell volume will be increased in all cases, with Te showing the highest increase in volume as it has the highest atomic radius.

### 3.2. Electronic Properties

Herein we will examine the electronic properties of the doped SnO_2_ by calculating the density of states (DOS) and partial DOS. The doping level is at approx. 2% per atom. The DOS and PDOS plots for the undoped cell are shown in Figure 2.

The band gap has been calculated as 3.6 eV using the PBE0 functional with a fairly ‘harder’ and quite accurate potential [24], included in the CASTEP code. The use of the Sn_d PAW pseudopotential, which includes 14 valence electrons and accounts for d-orbitals, yields a band gap for the undoped structure that is almost identical to the experimental value (3.62 eV) [25]. This approach appears to perform better than other computational works, which typically calculate the band gap as around 3.2 eV (with PBE0 or HSE06). Using the same code with standard pseudopotentials yields a band gap of 3.18 eV [9].

**Figure 3:**
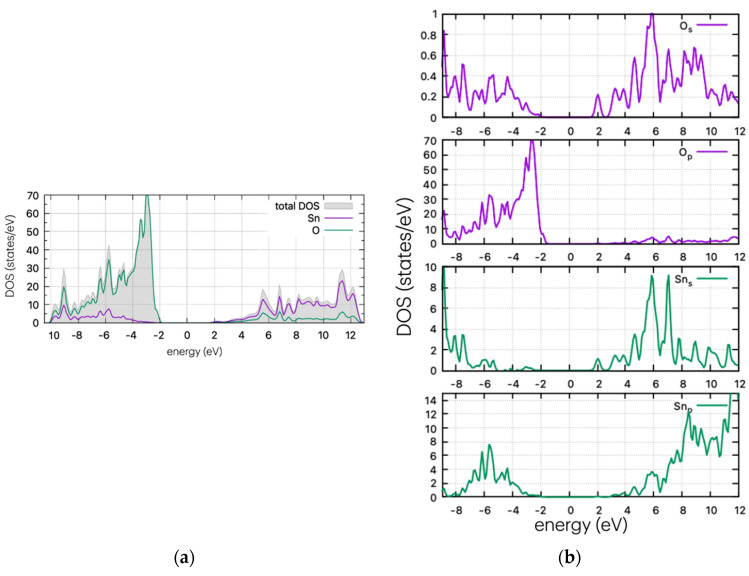
(**a**) DOS and (**b**) PDOS plots for the undoped SnO_2._

The DOS plots of the undoped structure are shown in Figure 3. The valence band of SnO_2_ is dominated by oxygen p-states, while the conduction band mostly comprises tin s- and p-states. The electrical conductivity of SnO_2_ is largely controlled by the movement of electrons in the tin d-orbitals.

**Figure 4:**
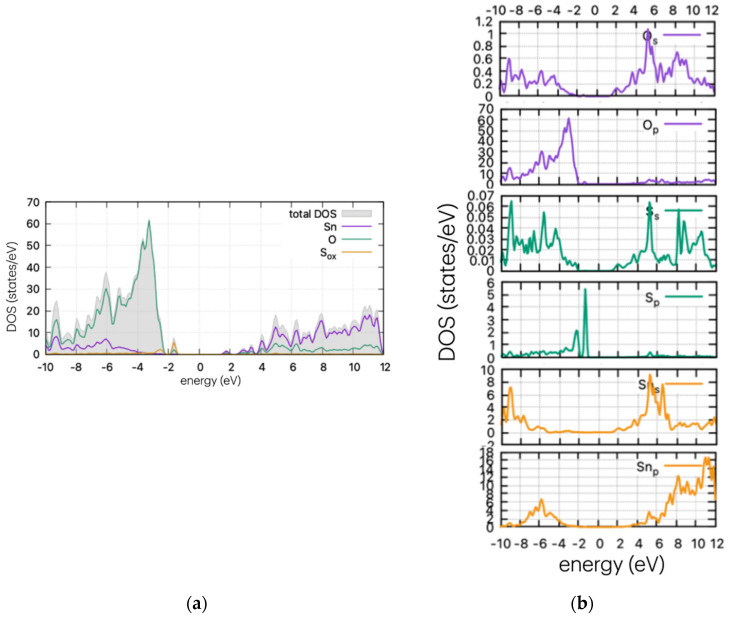
(**a**) DOS and (**b**) PDOS of S-doped SnO_2_ for oxygen substitutional position.

**Figure 5:**
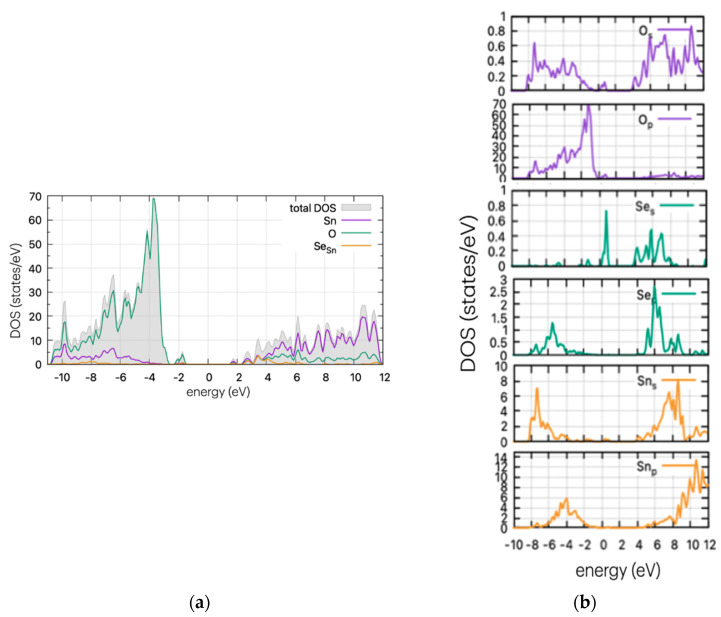
(**a**) DOS and (**b**) PDOS of Se-doped SnO_2_ for Sn substitutional position.

**Figure 6:**
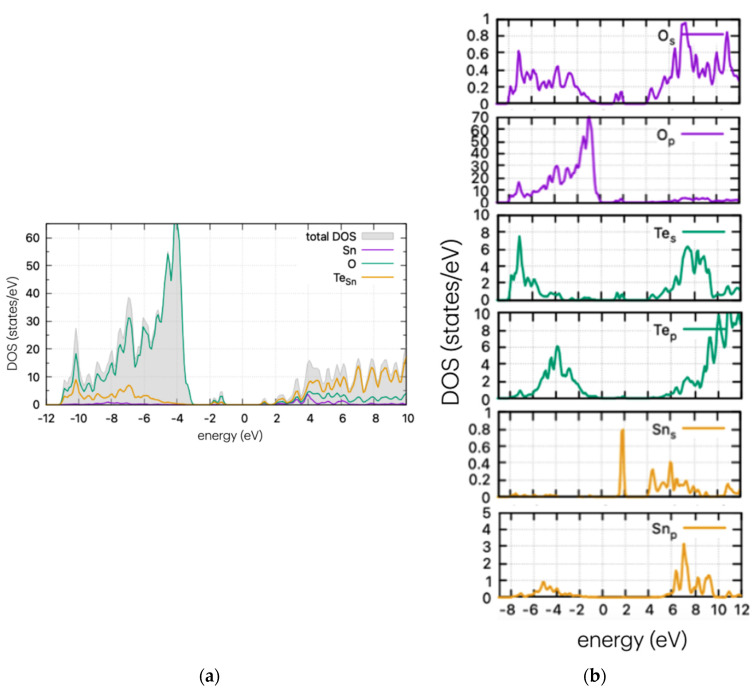
(**a**) DOS and (**b**) PDOS of Te-doped SnO_2_ for Sn substitutional position.

Using the same method as for the pristine supercell, we calculated the DOS of the doped structures. These are presented in Figure 4, Figure 5 and Figure 6 for the S, Se, and Te dopants, respectively.

As Figure 4 indicates, the insertion of S in the supercell leads to band gap states that are just above the valence band maximum (VBM). In the case of substitutional S in the oxygen position, the calculations show band tail-like behaviour and a reduction of the band gap at ~2.8 eV. The S peaks arise at 0.77 eV above the valence band maximum (VBM) for the oxygen substitutional case, and a second neighbouring peak is present for the interstitial case, at approx. 1.45 eV above the VBM. The band tail is expected to vary the optical properties of the material, producing a red shift in the optical absorption. This is examined in the next section. The formulated peak is due to the hybridisation of s-orbitals of S and O, as confirmed by partial DOS analysis (Figure 4b).

In Figure 5, the DOS of Se-doped SnO_2_ with Se replacing a Sn atom is presented. In this case (Se_Sn_), a Se peak is present as a band tail, reducing the band gap. The calculated results are very similar to the S case, which is to be expected as both dopants have four p-electrons in their outer shell. According to partial DOS analysis, these peaks are attributed to p-orbitals of Se and O. Se peaks are present at 0.77 eV above the VBM, and the band gap is at approximately ~2.7 eV, although this may vary slightly on the Gaussian smearing parameter (here set at 0.2). This decrease in value is consistent with the experimental work of Kumar et al. [26] who determined a decrease in the band gap (depending on the Se%), reaching a minimum of 2.9 eV and a red shift in the absorption spectrum.

In Figure 6, the calculated DOS of Te-doped SnO_2_ is shown for the Sn substitutional case. A strong and wide peak is evident below the middle of the band gap, at approx. 1.6 eV from the VBM, as well as a band tail nearing the conduction band edge. In contrast with the cases of S and Se doping, the calculations show that Te does not significantly reduce the band gap with the introduction of states near the band edges, but instead shows considerable mid-gap states. These can enhance photocatalytic processes by stabilising intermediate species or by providing additional reaction pathways. However, they can also reduce efficiency at high concentrations by increasing recombination rates. Therefore, understanding and controlling mid-gap states is important for optimising photocatalytic materials. For completeness, the DOS of all doping cases (interstitial and substitutional of Sn or O) are shown in Figure S1.

### 3.3. Optical Properties

Examining the optical properties can provide important information for comprehending the electronic structure of materials. These properties are derived from the complex dielectric function *ε*(*ω*) = *ε*_1_(*ω*) *+ iε*_2_(*ω*)*,* a quantity that characterises the material reaction to an external electric field. Using the CASTEP DFT code, we obtain the optical properties resulting from electronic transitions of the examined structures. We performed DFT calculations of the optical properties using LDA, PBE, and non-local PBE0 and HSE06 functionals. Hybrid functionals PBE0 and HSE06 yield, at low frequencies, a refractive index of approx. 1.52 for undoped SnO_2_, while non-local functionals LDA and PBE produce a refractive index of approx. 2 (Figure S2).

The refractive index of the undoped SnO_2_ may vary depending on the growth conditions and crystal structure of the sample. It has been reported to be about 1.9 [27] or 2.0 [28] at the incident photon wavelength of 550 nm (photon energy of 2.48 eV). Here we will present the DFT results obtained using the hybrid functional PBE0 (very similar to HSE06).

**Figure 7:**
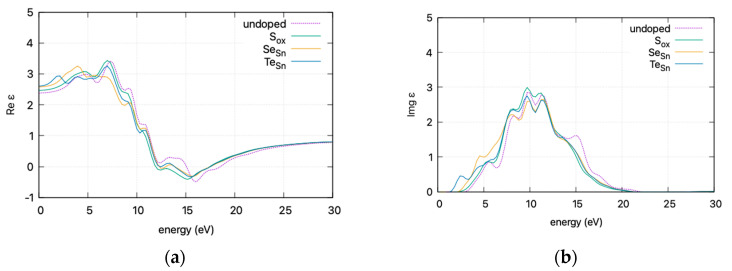
DFT calculated (HSE06) **dielectric function** vs. incident photon energy for doped and undoped SnO_2_: (**a**) real part *ε*_1_ and (**b**) imaginary part *ε*_2_. Graphs smoothed for visibility.

To present an overview of the impact of doping on the optical properties of the SnO_2_-doped system, the effect of doping on (a) the real part *ε*_1_ and (b) the imaginary part *ε*_2_ of the **dielectric function** is presented in Figure 7 (curves are smoothed for visibility).

**Figure 8:**
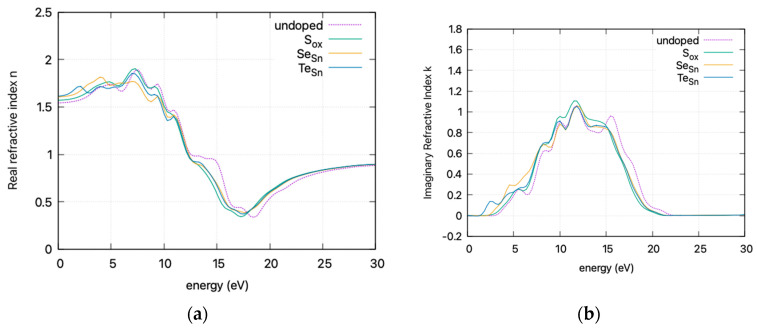
DFT calculated (HSE06) **refractive index** (smoothed curves) vs. incident photon energy for doped and undoped SnO_2._ (**a**) Real refractive index and (**b**) imaginary refractive index

**Figure 9:**
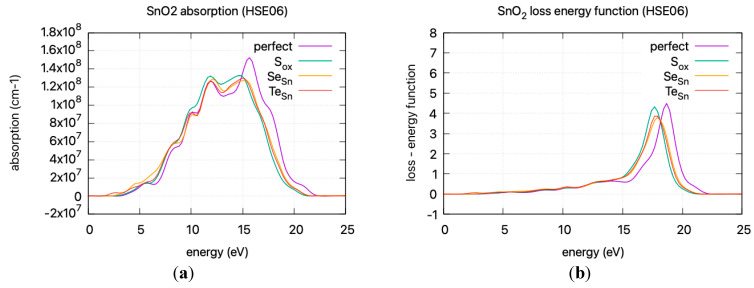
DFT calculated (HSE06) (**a**) absorption coefficient vs incident photon energy (**b**) loss energy function vs energy.

The calculated static dielectric function, *ε*₁(0), increases with doping, from 2.381 for undoped SnO_2_ to 2.466 for S doping, 2.576 for Se doping, and 2.604 for Te doping. A similar trend is observed for the *ε*_2_(*ω*) parameter in the low energy region, where the parameter exhibits a transition to non–zero values at lower energy levels for Te, Se, and S. This is consistent with the band gap calculations, as a greater static dielectric constant indicates a lower band gap [29]. At higher energy values, the *ε*(∞) has a maximum at almost ~4, consistent with wide band gap oxides. In Figure 8, the calculated refractive index is shown. Its values at low frequencies range from 1.54 for undoped SnO_2_ to 1.58 for S doping, 1.605 for Se doping, and 1.614 for Te doping. In Figure 9, the calculated absorption coefficients and loss energy function vs. energy are plotted. It can be observed that at higher energies (lower wavelengths), doping increases the absorbance of the film, making it less transparent.

The refractive index, calculated at low frequencies, varies from 1.54 for undoped SnO_2_ to 1.61 for Te doping. The static dielectric constant increases with doping. This increase is consistent with a lower band gap and is supported by band gap calculations. At higher energies, the *ε*(∞) approaches ~4, characteristic of wide band gap oxides. Additionally, the absorption coefficients and loss energy function indicate that doping results in increased absorbance, diminishing transparency at higher energies, leading to a red shift in the absorption spectrum.

## 4. Conclusions

The above findings can offer valuable guidance for tailoring SnO_2_ properties for applications ranging from photocatalysis to energy harvesting. The reduction of its bandgap, as demonstrated through S and Se doping, can enhance its suitability for such applications by increasing its capability to absorb light, particularly in the longer wavelengths of the spectrum (red shift), thus enabling the material to utilise a broader spectrum of sunlight. This can also have applications as an electron transfer layer (ETL) in photovoltaic devices, where a smaller band gap can improve band alignment with adjacent layers, which can significantly influence the overall performance of a solar cell. In the case of Te doping, the presence of energy levels in the middle of the bandgap could be beneficial in the context of photocatalytic or sensing applications as it provides a site where chemical reactions can take advantage of the trapped electrons. However, these mid-gap states can be detrimental for photovoltaic applications when Te-doped SnO_2_ is used as an ETL. Nevertheless, Te doping has theoretical potential for applications in photocatalysis, such as hydrogen production. In conclusion, this study provides additional insight into the effects of chalcogen doping of SnO_2_. The suitability of these doping techniques depends on various parameters, including overall device architecture, material compatibility, and performance requirements.

## Figures and Tables

**Table 1:** Calculated lattice constants and cell volume for the examined dopants at different positions.

SnO_2_	a (Å)	c (Å)	Vol (Å^3^)	ΔV (%)	E_formation_
perfect	4.818	3.236	75.121	n/a	n/a
S_Sn_	4.816	3.232	74.958	−0.218	5.43
S_ox_	4.833	3.240	75.681	0.745	3.95
S_int_	4.843	3.251	76.427	1.739	4.64
Se_Sn_	4.820	3.234	75.128	0. 009	3.90
Se_ox_	4.839	3.241	75.892	1.027	5.04
Se_int_	4.843	3.252	76.533	1.879	4.73
Te_Sn_	4.826	3.241	75.468	0. 462	2.88
Te_ox_	4.848	3.246	76.272	1.532	7.66
Te_int_	4.838	3.251	76.786	2.216	5.62

## Data Availability

The datasets used and/or analysed during the current study are available from the corresponding author upon reasonable request.

## Supplementary Materials

The following supporting information can be downloaded at: https://www.mdpi.com/article/10.3390/ma17163910/s1, Figure S1: The DOS of all doping cases (interstitial, substitutional of Sn or O). Figure S2: Comparison of refractive index calculated with LDA, GGA-PBE and hybrid functionals PBE0 and HSE06.

## References

[1] Batzill M., Diebold U. (2005). The surface and materials science of tin oxide. Prog. Surf. Sci..

[2] Li B., Zhou Q., Peng S., Liao Y. (2020). Recent Advances of SnO_2_-Based Sensors for Detecting Volatile Organic Compounds. Front. Chem..

[3] Khan M.M., Adil S.F., Al-Mayouf A. (2015). Metal oxides as photocatalysts. J. Saudi Chem. Soc..

[4] Dixon D., Ávila M., Ehrenberg H., Bhaskar A. (2020). Difference in Electrochemical Mechanism of SnO_2_ Conversion in Lithi-um-Ion and Sodium-Ion Batteries: Combined in Operando and Ex Situ XAS Investigations. ACS Appl. Energy Mater..

[5] Wu P., Wang S., Li X., Zhang F. (2021). Advances in SnO_2_-based perovskite solar cells: From preparation to photovoltaic applica-tions. J. Mater. Chem. A.

[6] Ma Y., Li L., Qian J., Qu W., Luo R., Wu F., Chen R. (2021). Materials and structure engineering by magnetron sputtering for advanced lithium batteries. Energy Storage Mater..

[7] Ko Y., Kim Y.R., Jang H., Chanyong L., Kang M.G., Jun Y. (2017). Electrodeposition of SnO_2_ on FTO and its Application in Planar Heterojunction Perovskite Solar Cells as an Electron Transport Layer. Nanoscale Res. Lett..

[8] Periyasamy M., Kar A. (2020). Modulating the properties of SnO_2_ nanocrystals: Morphological effects on structural, photolumi-nescence, photocatalytic, electrochemical and gas sensing properties. J. Mater. Chem. C.

[9] Kelaidis N., Zervos M., Lathiotakis N., Chroneos A. (2022). Vapor-Liquid-Solid Growth and Properties of One Dimensional PbO_x_ and PbO_x_/SnO_2_ (x = 1, 2) Nanowires for Energy Conversion and Storage. Mater. Adv..

[10] Sun C., Yang J., Xu M., Cui Y., Ren W., Zhang J., Zhao H., Liang B. (2022). Recent intensification strategies of SnO_2_-based photocatalysts: A review. Chem. Eng. J..

[11] Filippatos P.-P., Kelaidis N., Vasilopoulou M., Davazoglou D., Chroneos A. (2021). Impact of boron and indium doping on the structural, electronic and optical properties of SnO_2_. Sci. Rep..

[12] Filippatos P.-P., Sharma R., Soultati A., Kelaidis N., Petaroudis C., Alivisatou A.-A., Drivas C., Kennou S., Christopoulos S.-R.G., Davazoglou D. (2023). Optimization of the hydrogen response characteristics of halogen-doped SnO_2_. Sci. Rep..

[13] Filippatos P.-P., Kelaidis N., Vasilopoulou M., Davazoglou D., Chroneos A. (2021). Defect Processes in Halogen Doped SnO_2_. Appl. Sci..

[14] Xu K., Tian S., Zhu J., Yang Y., Shi J., Yu T., Yuan C. (2018). High selectivity of sulfur-doped SnO_2_ in NO_2_ detection at lower operating temperatures. Nanoscale.

[15] Ma L., Xu L., Xu X., Zhou X., Zhang L. (2016). One-Pot Hydrothermal Synthesis of Sulfur-Doped SnO_2_ Nanoparticles and their Enhanced Photocatalytic Properties. Nano.

[16] Yu J., Wang Y., Huang Y., Wang X., Guo J., Yang J., Zhao H. (2020). Structural electronic properties of SnO_2_ doped with non-metal elements. Beilstein J. Nan-otechnol..

[17] Zhao L., Gong X., Tao W., Wang T., Liu X., Liu F., Yan X., Wang C., Sun P., Lu G. (2021). Cationic Se-Doped SnO_2_ for Superior Acetone Sensing with a Moderate Baseline Resistance Rise. SSRN Electron. J..

[18] Georgieva B., Podolesheva I., Spasov G., Pirov J. (2014). Nanosized Thin SnO_2_ Layers Doped with Te and TeO_2_ as Room Temperature Humidity Sensors. Sensors.

[19] Graužinyte M., Goedecker S., Flores-Livas J.A. (2017). Computational screening of useful hole-electron dopants in SnO_2_. Chem. Mater.

[20] Lu P.F., Shen Y., Yu Z.Y., Zhao L., Li Q.Y., Ma S.J., Han L.H., Liu Y.M. (2012). Electronic structure and optical properties of antimony-doped SnO_2_ from first-principle study. Commun. Math. Phys..

[21] Castleton C.W.M., Höglund A., Mirbt S. (2009). Density functional theory calculations of defect energies using supercells. Model. Simul. Mater. Sci. Eng..

[22] Adamo C., Barone V. (1999). Toward reliable density functional methods without adjustable parameters: The PBE0 model. J. Chem. Phys..

[23] Momma K., Izumi F. (2011). VESTA 3 for three-dimensional visualization of crystal, volumetric and morphology data. J. Appl. Crystallogr..

[24] Lee M.-H. (1995). Advanced Pseudopotentials for Large Scale Electronic Structure Calculations With Application to A Study of Weakly Ordered Material—g-Al_2_O_3_. Ph.D. Thesis.

[25] Nagasawa M., Shionoya S. (1966). Exciton structure in optical absorption of SnO_2_ crystals. Phys. Lett..

[26] Kumar S., Chauhan P., Kundu V. (2016). Sol–gel synthesis, structural, morphological and optical properties of Se-doped SnO_2_ nanoparticles. J. Mater. Sci. Mater. Electron..

[27] Afify H.H., Momtaz R.S., Badawy W.A., Nasser S.A. (1991). Some physical properties of fluorine-doped SnO_2_ films prepared by spray pyrolysis. J. Mater. Sci. Mater. Electron..

[28] Manifacier J., De Murcia M., Fillard J., Vicario E. (1977). Optical and electrical properties of SnO_2_ thin films in relation to their stoichiometric deviation and their crystalline structure. Thin Solid Films.

[29] Ravichandran R., Wang A.X., Wager J.F. (2016). Solid State Dielectric Screening Versus Band Gap Trends and Implications. Opt. Mater..

