# Supplementary material for: Chalcogen Doping in SnO2: A DFT Investigation of Optical and Electronic Properties for Enhanced Photocatalytic Applications
Source: Materials (Basel). 2024 Aug 7;17(16):3910. doi: 10.3390/ma17163910 (PMC11355804; doi:10.3390/ma17163910)
Supplement: Supplementary file 1 [file materials-17-03910-s001.zip › materials-3099667-supplementary.pdf]

Supplementary Information

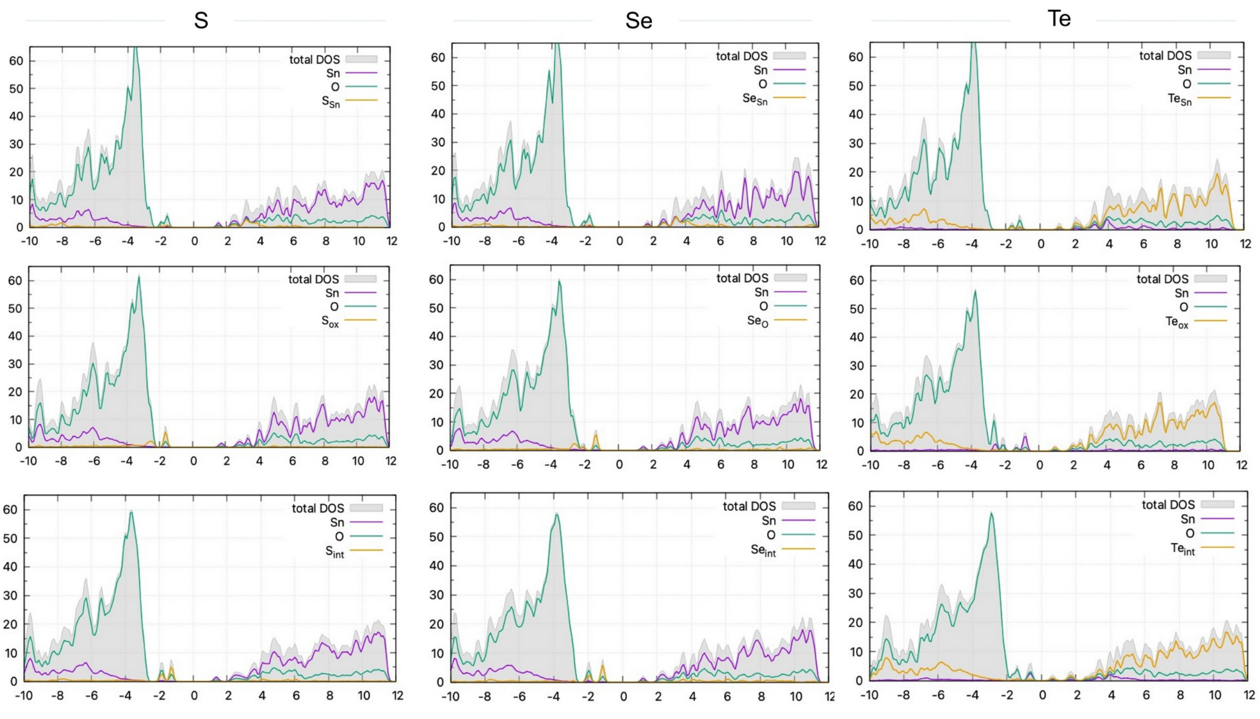

Figure S1. The DOS of all doping cases (interstitial, substitutional of Sn or O)

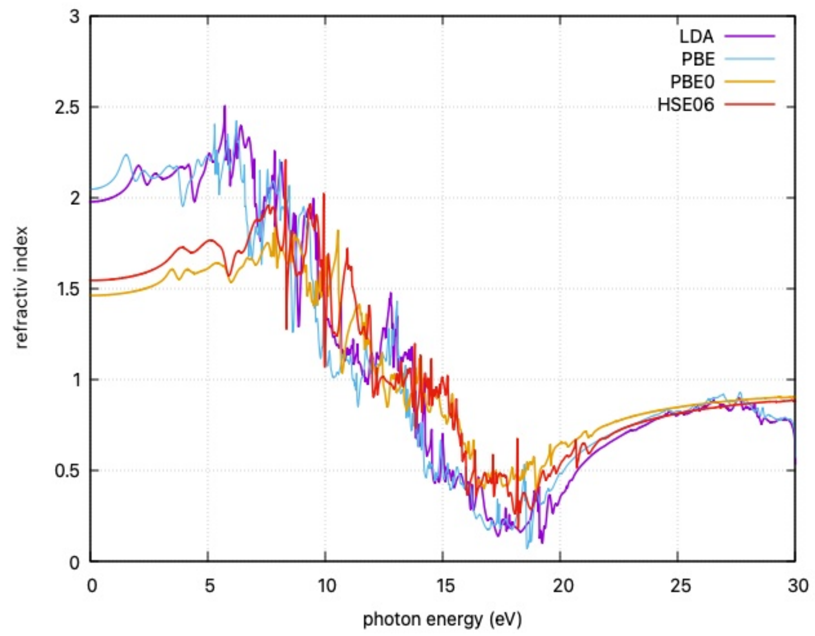

Figure S2. Comparison of refractive index calculated with LDA, GGA-PBE and hybrid functionals PBE0 and HSE06.
